# *Trypanosoma cruzi* vectors and reservoirs in Southern Sergipe

**DOI:** 10.1590/0037-8682-0740-2020

**Published:** 2021-04-12

**Authors:** Diana Matos Euzébio, Fábia Regina dos Santos, Daniel Matos Euzébio de Queiroz da Cruz, Ana Elisabeth Leal Varjão, Isabela Soares Costa, Guilherme Lopes Vasconcelos Manhães, Eduardo Melo Nascimento, Dalmo Correia, Angela Maria da Silva

**Affiliations:** 1 Universidade Federal de Sergipe, Programa de Pós-graduação Stricto Sensu em Ciências da Saúde, Aracaju, SE, Brasil.; 2 Universidade Federal de Sergipe, Hospital Universitário, Aracaju, SE, Brasil.; 3 Universidade Federal de Sergipe, Departamento de Medicina, Aracaju, SE, Brasil.; 4 Faculdade Pio Décimo, Programa de Graduação em Medicina Veterinária, Aracaju, SE, Brasil.; 5 Universidade Federal do Triângulo Mineiro, Programa de Graduação em Medicina, Uberaba, MG, Brasil.

**Keywords:** Chagas disease, Epidemiology, Serology, Triatominae

## Abstract

**INTRODUCTION:**

Chagas disease is a health problem that affects approximately 7 million people worldwide, according to the World Health Organization. Vector transmission is one of the most important routes in South and Central American countries. Between 2013 and 2019, municipalities of Sergipe sent 507 triatomines for analysis, unveiling the largest records found in the south in the villages of Poço da Clara, Alagoinhas and Pilões, and the municipality of Tobias Barreto. The high prevalence of infected vectors in these localities motivated this epidemiological study.

**METHODS:**

After educational lectures on the vectors and risks of the disease, a structured questionnaire was administered to identify areas and risk factors for transmission of the parasite. The data guided the collection of vectors and blood samples from domestic reservoirs.

**RESULTS:**

The studied region is considered endemic for triatomines infected by *Trypanosoma cruzi* with three species of vectors; the highest prevalence was *Panstrongylus lutzi* (54.83%), followed by *Triatoma pseudomaculata* (43.54%), and *Triatoma tibiamaculata* (1.61%). In the villages in this study, 100% of the vectors were found intradomically. The coexistence of residents with domestic animals was reported by 62.04% (255) of those surveyed. Forty-one small animals that were actively living with humans at home in the localities were evaluated serologically. No infection was observed in the domestic animals.

**CONCLUSIONS:**

There are favorable conditions for the domiciliation of triatomines in the evaluated locations, contributing to the risk of vectorial transmission of Chagas disease.

## INTRODUCTION

Chagas disease remains a global public health problem worldwide, and it is classified as one of the most neglected diseases by the World Health Organization, which indicates the need for its control^1^. The disease is caused by *Trypanosoma cruzi,* which is transmitted to humans via different routes. Triatomine insects are the vector route of transmission that prevails among other routes in several countries in South and Central America^1^. 

Environmental changes have led to the removal of the natural food sources of triatomines and, consequently, contributed to the vector’s adaptation to domestic and peridomestic areas, playing an important role in maintaining this route over time^2^.

In Brazil, a total of 5189 acute disease cases were reported to the Ministry of Health’s Notifiable Diseases Information System between 2001 and 2018, with 1,978 cases transmitted via oral routes, predominating over 1,839 vector-transmitted cases^3^. In the same period, a large number of incidences were registered with an unknown or unknown transmission route (1,327), while other more frequent routes were involved in fewer cases, including via transfusion (16), transplacental transmission (19), accidental transmission (4), and other unspecified unconventional forms (6).

The state of Sergipe had 99 acute disease cases from 2001 to 2018, with a predominantly vectorial transmission route^3^. Entomological data obtained from the Central Public Health Laboratory of Sergipe (LACEN) between 2013 and 2019 confirmed the presence of infected triatomine species in the villages of Poço da Clara, Alagoinhas, and Pilões in the municipality of Tobias Barreto, southern Sergipe, corroborating the risk of vector transmission^4^.

The relevance of these data motivated further research due to the identified favorable conditions for the maintenance of native triatomines present in the northeastern region of Brazil. These conditions can indicate *Triatoma infestans’* high capacity for domiciliation and the prevalence of risk factors associated with population exposure via vector transmission in the studied region^5^ ^,^ ^6^.

This study investigated the prevalence of triatomine species and infected domestic reservoirs to map the risk of transmission of human Chagas disease in the villages of Poço da Clara, Alagoinhas, and Pilões in Tobias Barreto, Sergipe, which are areas at risk of transmission.

## METHODS

Educational lectures were presented to the local population in the studied villages of Poço da Clara, Alagoinhas, and Pilões about vectors and risks of acquiring the disease. During these lectures, data on risk variables were collected through a structured questionnaire in concordance with the Free and Informed Consent Term (TCLE). The tabulation of the collected information showed areas with the presence of vectors that led to an active search and collection of insects, conducted in partnership with agents of the Endemic Service of Tobias Barreto-Sergipe, whilst following the guidelines recommended by Jurberg; Rodrigues and Moreira et al. (2014)^7^. In addition, we received spontaneous donations of triatomine specimens from the populations of the studied areas. 

Triatomine entomological data from LACEN, collected between 2013 and 2019, were used to identify the most prevalent species, vector infection rates, and localities with high vector incidence.

The sampling area was covered by using a 50 m transect, extending in all cardinal directions (north, south, east, and west) of a peridomycilium, and observing the natural environments such as the burrows, openings in the soil, bird nests, waste, tree trunks, henhouses, pigsties, and corrals.

Triatomines were collected in housing units through a manual search method, using a metal tweezer and flashlight to inspect dark openings/places. The investigation time was estimated to be 1 h per household collection.

**FIGURE 1:**
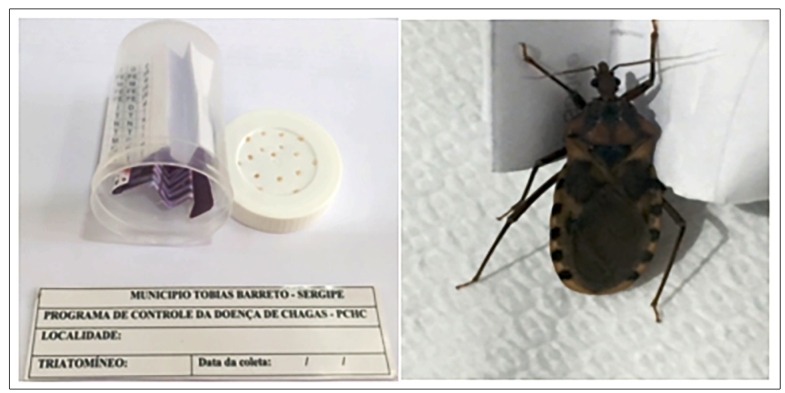
Packaging containing one male specimen of the triatomine *P. lutzi* captured by a villager in Poço da Clara - Sergipe, October 2019. **Source:** Euzébio, DM, 2019.

The recommendations of Galvão C and Paula AS (2014)^8^ were followed to capture, store, and transport the insects. The insects were stored in primary non-sterile plastic containers with perforated screwable lids (with at least ten holes), with a folded strip of paper inside, and identified by collection location area, date, and time, as shown in Figure 1.

Cardboard shoeboxes with small holes on the sides that were covered with a thin nylon fabric were used as secondary packaging. The primary plastic containers were placed inside these boxes, and compressed fabric was used to cushion and eliminate friction between the primary and secondary packaging.

The specimens were sent to LACEN for entomological and parasitological analyses, which were performed by an experienced professional. The classic parasitological method utilizing the abdominal compression technique and performing a microscopic evaluation of fresh and stained abdominal content on slides was used to observe trypomastigote forms of *Trypanosoma* spp. The results were reported to the Tobias Barreto's Health Secretary as a guide for the adoption of the necessary disease preventive measures in the studied localities.

Domestic animals, considered domestic reservoirs, were selected in the evaluated areas for serological analysis using questionnaires, accessing household information and results of positive triatomine tests for *T. cruzi*. Samples were collected via a peripheral venous puncture by a veterinarian, a collaborator in the study, and endemic agents of the municipality of Tobias Barreto, Sergipe. All study participants (animal owners) were informed and signed the TCLE agreement. After collection, animals were released in their environment immediately and observed for clinical conditions until they spontaneously resumed their activities. The collected blood samples were processed in the laboratory of the Hospital Universitário de Sergipe. Tests with different antigenic preparations were used to evaluate the presence or absence of antibodies against *T. cruzi* in the studied samples. Indirect immunofluorescence was performed manually on slides using a technique similar to human immunoassay and automated chemiluminescence microparticle immunoassay tests for the qualitative detection of antibodies against *T. cruzi*, following the manufacturer’s recommendations.

The results obtained were stored in an Excel spreadsheet and analyzed using Epi Info version 7.1.4, R version 3.6.3, and GeoDa version 1.14. Odds ratios were calculated between the factors of disease exposure and individuals bitten by the vector. Fisher’s exact test was used to compare *T. cruzi* positivity among species of prevalent triatomines collected in Sergipe. GeoDa software was used with the univariate global Moran index and the pseudo-significance test for non-normal distributions to analyze the spatial autocorrelation of triatomines in the study.

### Ethical considerations

The study was approved by the Ethics and Research Council under opinion number 1.486.038, considering the guidelines and regulatory standards for research involving human beings contained in Resolution 466/2012 of the National Health Council and the Brazilian Guidelines for Care and Use of Animals for Scientific and Didactic Purposes, Brasília/DF - 2013.

## RESULTS

A total of 507 insects were received from 28 municipalities in Sergipe between 2013 and 2019, represented by 455 adults and 52 nymphs. The studied region is considered endemic for triatomines infected by *T. cruzi* with three species of vectors; the highest prevalence among these vectors was *Panstrongylus lutzi* (54.83%), followed by *Triatoma pseudomaculata* (43.54%) and *Triatoma tibiamaculata* (1.61%). The index of infection by *T. cruzi* from *Triatominae* species collected in Sergipe is shown in Table 1. The predominant species of *T. cruzi* in Sergipe were *T. pseudomaculata*, *P. lutzi*, and *Triatoma brasiliensis*, comprising 83.04% of all samples in the state of Sergipe. Only one specimen of the species *Panstrongylus geniculatus* was collected from the peridomycilium site in the municipality of Tobias Barreto. This species is considered essentially wild and not frequently found in the state of Sergipe, although it can easily adapt to various environments and different biomes^8^. There were no records of *T. infestans* between 2013 and 2019.

**TABLE 1:** Index of infection by *T. cruzi* from Triatominae species collected in Sergipe from 2013 to 2019.

	Species		Positivity by total		Fisher’s exact test
	Positivity by species		of other species		**(*p*-value)**
	Negative	Positive	Negative	Positive	
*Panstrongylus geniculatus*	0	1	468	37	0.144
*Panstrongylus lutz*i	129	22	340	16	< 0.01
*Triatoma brasiliensis*	72	4	397	34	0.635
*Triatoma melanocephala*	5	1	464	37	0.374
*Triatoma pseudomaculat*a	235	9	236	29	1
*Triatoma tibiamaculata*	16	1	453	37	1

**Source**: Central Public Health Laboratory of Sergipe, Entomology Service, Sergipe (2019).

**FIGURE 2:**
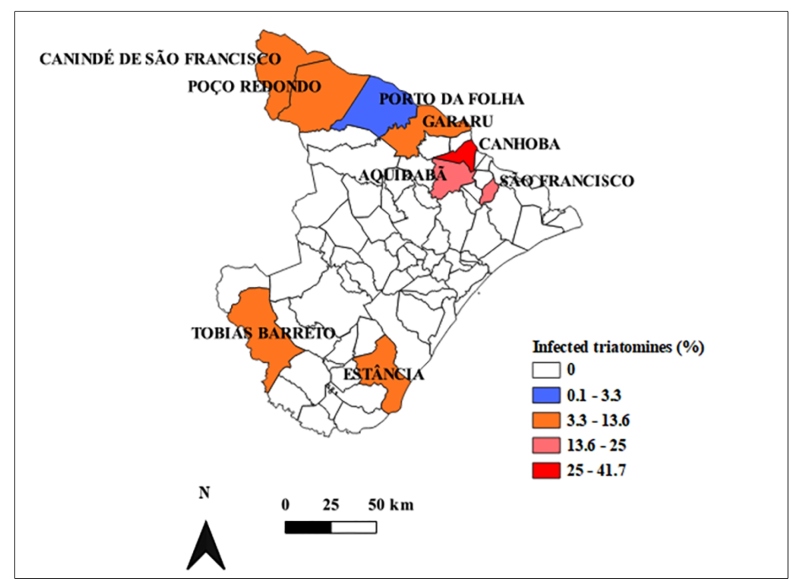
Entomological indexes of infected triatomines analyzed in the municipalities of Sergipe, Brazil, from 2013 to 2019. **Source**: Central Public Health Laboratory of Sergipe, Entomology Service, Sergipe (2019).

The highest rates of natural infection of triatomines were observed in the municipalities of Canhoba (41.66%), Santana do São Francisco (20.0%), Aquidabã (33.33%), and Tobias Barreto (13.17%), as shown in Figure 2.

The univariate global Moran index and pseudo-significance test of the percentage of infected triatomines (I = 0.018; pseudo *p-value* = 0.212), and the absolute number of triatomines (I = 0.066; pseudo *p-value* = 0.098) were calculated using GeoDa 1.14.0, demonstrating the existence of spatial clusters in both cases.

The villages of Poço da Clara, Alagoinhas, and Pilões sent 15.47% (62) of the Sergipe triatomines for analysis. The highest prevalence was found in Poço da Clara, followed by Pilões. The natural infection rate in the insects evaluated at these locations was 16.12% (Table 2).

**TABLE 2:** Captured specimens and natural infection rate of triatomines in the villages of Poço da Clara, Alagoinhas, and Pilões in Tobias Barreto, Sergipe, from 2013 - 2019.

Research Villages/species	Peridomicile	Intradomicile	Males		Females		Natural Infection Rate%
			Positive	Negative	Positive	Negative	
**Alagoinhas**	0	14	1	12	1	0	16,66
*Panstrongylus lutzi*	0	10	0	9	1	0	-
*Triatoma pseudomaculata*	0	4	1	3	0	0	-
**Pilões**	0	21	5	16	0	1	23,52
*Panstrongylus lutzi*	0	8	3	5	0	0	-
*Triatoma pseudomaculata*	0	12	2	9	0	1	-
*Triatoma tibiamaculata*	0	1	0	1	0	0	-
***Poço da Clara***	0	27	1	25	1	1	17,39
*Panstrongylus lutzi*	0	16	0	15	2	0	-
*Triatoma pseudomaculata*	0	11	1	10	0	1	-
**Overall Total**	0	62	7	53	3	2	16,12

**Source**: Central Public Health Laboratory of Sergipe, Entomology Service, Sergipe (2019).

Among the 62 captured specimens, 10 were identified as positive for *Trypanosoma* spp.; all specimens were in the adult stage and found in an intradomicile site; *P. lutzi* species predominated, followed by *T. pseudomaculata*. No nymphs were found in the studied villages.

**FIGURE 3:**
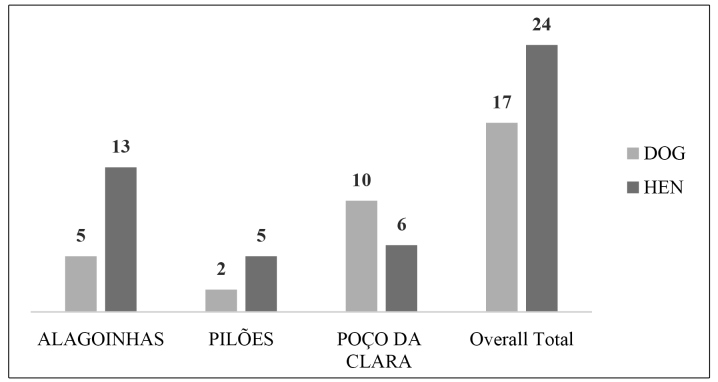
Assessment of *Triatominae* food sources in the villages of Alagoinhas, Pilões, and Poço da Clara in 2019. **Source:** Euzébio, DM, 2019.

The coexistence of residents with domestic animals was reported by 62.04% (255) of those surveyed. Forty-one small animals that were actively living with humans at home in the localities where the study participants reported finding triatomines were evaluated serologically. The distribution of the results for the evaluated animals is shown in Figure 3. No *T. cruzi* serologies were identified in the evaluated animals. 

## DISCUSSION

The studied region is considered endemic for triatomines infected by *Trypanosoma* spp*.* with three species of vectors; the highest prevalence among these vectors was *P. lutzi* (54.83%), followed by *T. pseudomaculata* (43.54%) and *T. tibiamaculata* (1.61%). *P. lutzi* has a high rate of natural infection and good dispersion ability during flight. However, both *P. lutzi* and *T. pseudomaculata* have reduced vectorial capacity and competence with low metacyclogenesis^9^.

In line with the work of Caranha et al. (2006)^10^, *P. lutzi* was the species with the most significant infection rate by *Trypanosoma* spp*.* in Poço da Clara, Pilões, and Alagoinhas. The observed low rates of vector transmission to humans and domestic reservoirs associated with this species may be explained by the low metacyclogenesis.

The study by Candido et al. (2019)^11^ conducted in the state of Ceará showed *that T. pseudomaculata*, *T. brasiliensis*, and *P. lutzi* as the most prevalent species, the latter mostly found in homes, corroborating the data found in our study.

Studies have described the presence of adult insects of this species in the home without colonization associated with domestic animals, corroborating the data found in the study region^12^.

 Despite the low capacity for metacyclogenesis among some species found in this study, the Chagas Disease Prevention and Control Program^13^ recommends the application of chemical control in households where at least one live adult triatomine or colony is found among some species, including *Panstrongylus megistus*, *T. brasiliensis*, *T. maculata*, *T. pseudomaculata*, *Triatoma rubrovaria*, and *Triatoma sordida*. Some of these species are found in Sergipe.

Studies have shown a reduction in parasitemia in vectors with less metacyclogenesis in the absence of a new repast^9^; these levels of parasitemia tend to rise when an opportunity for a new repast is presented. Nymphs in stages third and fourth showed higher mortality than adult insects after prolonged periods of fasting, which corroborates the observation of higher numbers of infected insects in adult stages than nymphs in the studied area^14^.

Some authors have described *T. pseudomaculata* and *P. lutzi*, which were previously found exclusively in wild environments, as having behavioral adaptations that allow them to coexist with other species, leading to new food source opportunities^8^ ^,^ ^10^ ^,^ ^14^. Our results are in agreement with the aforementioned authors all specimens collected in the studied villages were found inside houses, suggesting possible colonization.

Our data showed that 100% of the collected vectors in the studied villages were adults; however, it was not possible to observe colonies in shelters investigated during visits to home units, and capture attempts occurred during the day without using insecticides to dislodge vectors. However, the constant presence of vectors in these homes suggests a close relationship with the search for food sources by the vectors.

Silveira and Dias (2011)^15^ described the visitation of *T. cruzi* vectors in homes without domicile colony installations as responsible for the transmission of Chagas disease, likely because these species are becoming increasingly frequent in the northern regions of the country that have human installations in forest regions.

Precarious houses with walls built using the rammed-earth construction technique are favorable for the domiciliation of vectors. Constructions of this type create humid and hot environments that tend to crack over time, which represent favorable conditions for the installation of colonies of *T. cruzi* ^8^ vectors. The impact of this type of construction near areas with environmental devastation and little or no food source is a driver for *T. cruzi* vector insects to move from their natural ecotopes and guarantee their survival. *P. lutzi* in the Northeast region has shown a high degree of adaptation to other ecotopes, favoring domiciliation^8^ ^,^ ^16^ ^,^ ^17^.

Data from 1975 to 1983 in the Brazilian territory showed a higher presence of *P. lutzi* in the state of Pernambuco compared with other states, with values close to the national total in several years. The results of our study demonstrate the epidemiological importance of adult insects of the *P. lutzi* species inside homes in the studied villages, indicating the possibility of domiciliation, even though no eggs or nymphs were found. The highest rates of natural infection that were found in the study sites that were linked to *P. lutzi* was 20.58%, which is in concordance with the percentage of 29.4% reported by the Ministry of Health (2015)^18^. There was a statistically significant difference in the prevalence of *P. lutzi* compared with other species (*p* < 0.001), indicating the risk of vector transmission.

The findings of this study show that of the 57.27% of insects that were predominant in the state of Sergipe, 44.63% were intradomicile. Costa et al. (2003)^19^ and Sarquis et al. (2004)^5^ reported that this species is frequently found in domiciles in almost all states of the Brazilian semiarid region, leading to them being among the predominant vectors in the Caatinga, even being found in the transitional area of the agreste in the semiarid northeast. 

Our results show that *T. pseudomaculata* is the second most prevalent intradomicile species in the studied villages. According to the classification of several studies, *T. pseudomaculata* is described among autochthonous vectors that continuously colonize the home environment^9^ ^,^ ^19^ ^,^ ^20^. This situation increases contact through exposure and, therefore, increases the risk of disease. However, their long repast and defecation time do not characterize this species as a good vector for *T. cruzi* ^21^.

A study by Gonçalves et al. (1997)^21^ evaluated the biological aspects of *T. pseudomaculata* in the laboratory, including the time interval between repast and defecation, which reached approximately 10 minutes. Transmission of *T. cruzi* occurs when the feces of infected insects touch lesioned mucous membranes. Therefore, extended intervals between repast and defecation can reduce the risk of transmission; this is because the insect might have moved away from the spot where the bite occurred before defecating close by. Short intervals between repasting and defecation represent an important factor for parasite transmission, increasing the risk of exposure to infected insect vectors^8^.

*T. tibiamaculata* was found in 1.58% of the intradomicile sites in the village of Pilões. This vector is considered essentially wild, with an eventual presence in other environments, and presents with reduced vector competence^8^ ^,^ ^16^. According to Galvão C and Paula AS (2014)^8^, the species was found to be naturally infected by *T. cruzi* parasites when caught from epiphytic bromeliads and near or in marsupial nests.

Although the behavior described for this species is wild, all samples captured in Sergipe were found in intradomicile environments, which is in line with an epidemiological descriptive survey conducted in the state of São Paulo between 2010 and 2012, which demonstrates the adaptability of these vectors^22^.

The univariate global Moran index and the pseudo-significance test of the percentage of infected triatomines showed no spatial clusters in the studied villages with *T. cruzi-infected* triatomines. The prevalent species in the studied localities present characteristics that demonstrate low infective power, such as the longer repast and defecation times of *T. pseudomaculata*, and low metacyclogenesis rates of *P. lutzi*, which justifies the low transmission rate of disease to humans in the studied region. However, the high capacity of domiciliation of the mentioned species causes concern from an epidemiological point of view and requires monitoring of *T. cruzi* ^8^invertebrate vectors in endemic areas. The studied localities demand entomological vigilance because they present wild species in intra-and peridomicile environments.

Nymphs found in intradomicile environments have great epidemiological relevance, suggesting possible colonization; this is a widely used indicator in the planning of actions to control Chagas disease in humans^23^ ^,^ ^13^. The analysis of the stages of the insects that were sent by several municipalities in the state of Sergipe showed that 89.74% of them were in the adult phase and 10.26% were nymphs, indicating the maintenance of the vector’s life cycle in several regions of the state.

The maintenance of the wild disease cycle is related to the association between triatomines and small wild animals, which are the natural food sources of these vectors and reservoirs for *T. cruzi*. According to Villela et al. (2009)^24^, the existence of wild outbreaks of *T. cruzi* vectors and potential reservoirs in endemic areas favors the continuous occurrence of infestations in home units for long periods.

Serological surveillance of domestic reservoirs is a recommended practice in home units where vectors of *T. cruzi* are found without the location of peridomiciliary colonies in endemic areas^25^. Since domestic animals may represent a timelier source of food for these vectors than humans and considering that chronic Chagas disease infection in humans may remain undetected for decades until diagnosis, serological surveillance of these animals is essential to identify areas at risk of transmission^25^ ^,^ ^26^.

Studies have shown that dogs can maintain high rates of parasitism for an extended period of time^25^ ^,^ ^26^. Several studies recommend serological monitoring of these animals as sentinels for the presence of the parasite in areas at risk of disease transmission^25^ ^,^ ^26^ ^,^ ^27^.

A study conducted in dog shelters in Louisiana in the United States^25^ demonstrated high serological positivity for *T. cruzi*. This species’ intimate coexistence in domestic environments and their good competence as reservoirs that maintain high levels of parasitemia in the blood make them an important link in the domestic vector transmission chain of the disease in triatomine endemic areas^28^.

The study by Bezerra et al., (2018)^29^ highlights the breeding of chickens and goats in Ceará as an important element for the approximation of triatomines; this can be done by identifying triatomines DNA in the animals’ intestinal contents. The findings of our study indicate similar conditions of increased opportunities for vectors to find food, demonstrated by the high percentages of triatomines’ prevalence in sites with coexisting domestic animals and humans, increasing the risk of infection.

Even without positive serological results for *T. cruzi* in the evaluated domestic animals, Borges et al. (2005)^30^ pointed out that the increased opportunity of food sources can contribute to the growth of triatomine populations and colonization in the home environment and areas of human settlements.

A survey at the Nucleus of Endemics of Tobias Barreto, which referred to the localities of Poço da Clara, Alagoinhas, and Pilões^4^, showed that the risk of infection by Chagas disease is high in the studied villages due to the endemicity of the triatomines that were infected with *T. cruzi.* The survey also revealed that the number of exposures through contact with vectors indicated by the participants’ responses may favor the transmission of the parasite.

Domestic reservoirs infected with *T. cruzi* can contribute to the maintenance of infection in humans, representing an important link in the chain of transmission in the studied region.

The conclusion is that there is vector transmission in the villages of Poço da Clara, Alagoinhas, and Pilões, which are in the rural area of the Sergipe state. Although the percentage of infection in humans in this area is reported to be lower than the estimated national incidence, the prevalence of *T. cruzi* vectors was verified as displaying a high risk of domiciliation, also demonstrating that the disease is underreported in this region. Moreover, the high incidence of coexistence with domestic animals in the region represents an opportunity for the domiciliation of wild vectors.

*T. pseudomaculata* and *P. lutzi* are among the most prevalent species in Sergipe and have a high capacity for domiciliation, representing the main vectors in the transmission of the disease and ratifying the risk of vector transmission.

Given the number of infected triatomines found in the study, the favorable conditions for their approximation, and given the risk of transmission of *T. cruzi*, the monitoring of wild animals in the region is suggested as a future preventive action to mitigate the risk of increased cases of Chagas disease.

The results obtained in this study confirm that Chagas disease is not being diagnosed, and vector transmission is not being interrupted in the studied region, demonstrating the need for greater emphasis on policies for the prevention and control of this disease.
